# A Sensitive and Stable Surface Plasmon Resonance Sensor Based on Monolayer Protected Silver Film

**DOI:** 10.3390/s17122777

**Published:** 2017-11-30

**Authors:** Guiqiang Wang, Chunnan Wang, Rui Yang, Wenlan Liu, Shuqing Sun

**Affiliations:** 1 Shenzhen Key Laboratory for Minimal Invasive Medical Technologies, Institute of Optical Imaging and Sensing, Graduate School at Shenzhen, Tsinghua University, Shenzhen 518055, China; wgq14@mails.tsinghua.edu.cn (G.W.); wcn16@mails.tsinghua.edu.cn (C.W.); r-yang15@mails.tsinghua.edu.cn (R.Y.); 2 Department of Physics, Tsinghua University, Beijing 100084, China; 3 The Central Laboratory, The First Affiliated Hospital of Shenzhen University, Shenzhen Second People’s Hospital, Shenzhen 518035, China; wlliu@szu.edu.cn

**Keywords:** silver film, surface plasmon resonance, chemical stability, self-assembled monolayer (SAM)

## Abstract

In this paper, we present a stable silver-based surface plasmon resonance (SPR) sensor using a self-assembled monolayer (SAM) as a protection layer and investigated its efficiency in water and 0.01 M phosphate buffered saline (PBS). By simulation, silver-based SPR sensor has a better performance in field enhancement and penetration depth than that of a gold-based SPR sensor, which are 5 and 1.4 times, respectively. To overcome the instability of the bare silver film and investigate the efficiency of the protected layer, the SAM of 11-mercapto-1-undecanol (MUD) was used as a protection layer. Stability experiment results show that the protected silver film exhibited excellent stability either in pure water or 0.01 M PBS buffer. The sensitivity of the silver-based SPR sensor was calculated to be 127.26 deg/RIU (refractive index unit), measured with different concentrations of NaCl solutions. Further, a very high refractive resolution for the silver-based SPR sensor was found to be 2.207 × 10^−7^ RIU, which reaches the theoretical limit in the wavelength of 632.8 nm for a SPR sensor reported in the literature. Using a mixed SAM of 16-mercaptohexadecanoic acid (MHDA) and a MUD layer with a ratio of 1:10, this immunosensor for the rabbit immunoglobulin G (IgG) molecule with a limit of detection as low as 22.516 ng/mL was achieved.

## 1. Introduction

The surface plasmon wave was first reported by Wood in 1902 [1], and more than 60 years later, Kretschmann [2] and Otto [3] realized the surface plasmon resonance with the method of attenuated total reflection in 1968. Then, over the last decades, label-free, real-time and ultra-sensitive surface plasmon resonance (SPR) sensor has been rapidly developed for the detection of small and large molecules based on the monitoring of refractive index change of surrounding media [4,5,6,7], and it has been widely used in the field of drug screening [8,9,10] and other biomedical areas [11]. Practically, the stability of biosensor chips is significant for repeatable results. To meet this requirement, gold film is usually used to excite the SPR mode due to its chemical stability. 

Silver is another material which can be used for SPR sensors. Silver-based SPR sensors exhibit higher sensitivity and sharper reflectivity spectrum than those of gold film. The limit of detection (LOD) or resolution would be significantly improved when silver film is used as the excitation film. However, the poor chemical stability limits its application. So far, methods to protect silver film from oxidation and sulfuration mainly involve the use of bimetallic silver/gold layers [12,13,14,15,16,17], deposition of dielectric thin films [18,19,20,21,22,23,24] or polymers [25], and the use of self-assembled monolayers (SAM) [26,27,28,29,30,31,32]. In the structure of silver/gold film, the silver was protected by an overlay of ca.15 nm-thick gold film [14], which is directly connected with the air or the sample solution. However, a thicker gold film could provide a better protection, and the influence of the presence of thin gold film on the SPR spectrum would be enlarged [16]. Another approach is the use of dense oxide thin films or polymers [25] and oxide film are the common protection layers, such as TiO_2_ [21] and SnO_2_ [19,20]. Those oxide film could effectively protect the silver film and also open a new functionalization method compared with pure metal film. In addition, thin films of carbon such as grapheme [18] or amorphous silicon-carbon alloys such as a-Si_0.63_C_0.37_ film [23] were also used in the silver-based SPR sensor, and allowed the convenient functionalization with biomolecules via C-C or Si-C bonds. Further, a base SiOxCyHz layer could also be deposited on silver film as a protective layer by plasma enhanced chemical vapor deposition (PECVD) [24].

Although the above methods could effectively protect the silver film, the procedures are usually complex or time consuming, and the protective layers generally affected the performance of silver-based SPR sensor adversely. The thickness of SAM is usually several nanometers and can preserve the optical properties of silver film effectively [17]. In addition, the use of SAM is a more convenient and facile process, which makes it easier for practical applications. Many researches mainly focused on monitoring the formation of SAM film on metal film by changing the concentration of the solution [28,31,33] or the chain length of sulfhydryl molecules through SPR sensors [29]. The red shift of the dip of SPR spectrum linearly respects to the SAM thickness or the chain length of sulfydryl molecules. Thus, the concentration of thiol molecules in solution could be detected. With the adsorption of cysteine on silver nanocubes, the detection level is down to 10 μM, and the SAM of cysteine is also a protecting layer [27]. Further, the adsorption of fewer than 60,000 1-hexadecanethiol molecules on a single silver nanoparticles, that is, 100 Zeptomole, is also achieved [30]. However, few reports focus on the performance of silver film-based SPR sensors using SAM as a protection layer. 

Herein, we report the use of SAM as a protection layer for silver film and investigate its efficiency. Since the hydroxyl group resists nonspecific protein adsorption and the carboxylic acid end groups enable subsequent derivatization of the SAM [34], carboxylic acid terminated thiol molecules with longer alkyl chain were further used in the mixed SAMs for the immobilization of biomolecules for highly sensitive detection of protein, rabbit IgG. Using this configuration, the index resolution for the silver-based SPR sensor was obtained to be 2.207 × 10^−7^ RIU and the limit of detection as low as 22.516 ng/mL was achieved for rabbit IgG molecule.

## 2. Materials and Methods 

### 2.1. Materials

K9 glass substrates (22 × 16 × 0.5 mm^3^) were bought from Haian Country Xiongying Photoelectricity Equipment Factory (Nantong, China). 11-Mercapto-1-undecanol (97%, MUD), 16-Mercaptohexadecanoic acid (90%, MHDA) and N-(3-Dimethylaminopropyl)-N-ethylcarbodiimide hydrochloride (98%, EDC) were purchased from Sigma Aldrich. Immunoglobulin G (IgG) protein molecules, rabbit IgG and goat anti-rabbit (GAR) IgG were acquired from Beijing Biosynthesis Biotechnology Co., Ltd. (Beijing, China). Bovine Serum Albumin (BSA) was obtained from Biosharp Biotech Co., Ltd. (Hefei, China). Sodium chloride (NaCl) was purchased from Alfa Aesar (Tianjin, China). Others were obtained from Shenzhen Senke Instruments Co., Ltd. (Shenzhen, China). All the reagents were used as received without further purification. The 18.2 MΩ/cm Milli-Q water was used in all of our experiments. 0.01 M PBS buffer (PH 7.4, 8 mM Na_2_HPO_4_, 2 mM KH_2_PO_4_, 136 mM NaCl, 2.6 mM KCl) and 0.01 M PB buffer (PH 7.4, 8 mM Na_2_HPO_4_, 2 mM KH_2_PO_4_) were prepared with Milli-Q water and filtered with 0.2 μm Millex Syringe Filters. The PB buffer was used in the detection of rabbit IgG molecule.

### 2.2. Methods

#### 2.2.1. Sputtering Silver or Gold Film on the K9 Substrate

Silver or gold film was prepared by magnetron sputtering (SAJS450, Shenyang Tengao Machinery Manufacturing CO., Ltd., Shenyang, China) of silver onto a K9 glass substrates cleaned with piranha solution (note that it is very dangerous; please be careful during operation). Chromium (Cr) was used as an adhesion layer between the metal film and the substrate and its thickness was set at ca. 2 nm. Both the thicknesses of metal films are about 50 nm and the working condition of the magnetron sputter is 0.50 Pa (silver) or 0.65 Pa (gold), 50 W with a reverse bias voltage of 150 V.

#### 2.2.2. Formation of SAM and Mixed SAMs on the Metal Film

The deposited silver film chip was immediately immersed into the alcoholic solution of MUD (4 mM MUD) for 2 h to form an SAM, and was then washed with plenty of alcohol and water. After drying with a flow of N_2_, the chip was either subjected to optical experiments or stored at ambient condition for the stability test. Bare silver film without any modification was used as reference chip for stability comparison. The formation of mixed SAMs of MUD and MHDA on silver or gold film was completed using a binary mixture of alcoholic solution of MUD (5 mM) and MHDA (0.5 mM) to replace alcoholic MUD solution with otherwise identical procedures. The mole ratio of MHDA in the mixed SAMs was set to 0.1 since the SPR sensor obtains its maximum response at this condition [34].

#### 2.2.3. Angular Spectrum Setup of Silver-Based SPR Sensor 

**Figure 1:**
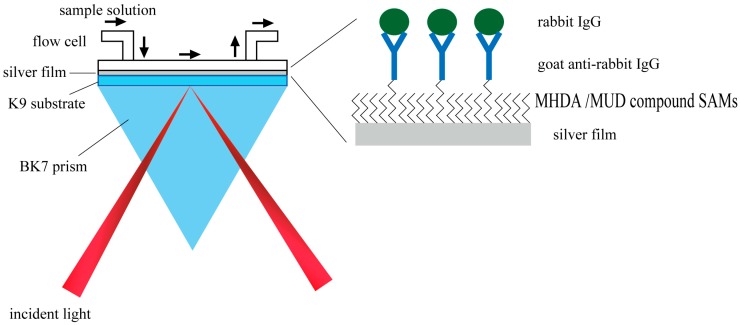
Schematic diagram of angular spectrum setup.

The anti-IgG molecule detection was carried out based on a homemade angular spectrum setup. As shown in Figure 1, the light source we used was a red LED light (Osram LR W5AP, 5 W) combined with a bandpass filter (central wavelength 632.8 nm, full width half maximum 3 nm). Transverse magnetic (TM) mode was obtained by a linear polarizer and different incident angles were acquired by a cylindrical lens. Finally, the angular spectrum was captured by a CCD (Charge Coupled Device) camera (QICAM Fast, 1394 × 1040 pixels, QImaging, Surrey, BC, Canada).

#### 2.2.4. Stability Test for the MUD SAM-Protected and Bare Silver Films 

The stability tests include two aspects, continuous stability test and long-term storage stability test. The angular spectrum was obtained by another homemade angular spectrum SPR sensor, and more details could be found in our previous works [35,36,37,38]. In short, the source light is a HeNe laser and the angle is accuracy controlled and changed with electronic rotators and translation stage. Further, the scanning angle is from 60 to 76 deg, which is large enough to cover the total internal reflection (TIR) angle and the resonance angle of silver-based SPR sensor in our stability test. 

The continuous stabilities of MUD SAM-protected silver and bare silver film were first tested in both water and 0.01 M PBS buffer, which were pumped into a flow cell ($16\times 2\times 0.15{\;\mathrm{mm}}^{3}$) by a syringe pump with a speed of 100 μL/min, and their angular spectra were collected every 5 min, lasting for 30 min.

In the long-term storage stability test, silver films were stored in ambient atmosphere and the angular spectra were obtained periodically. The shifts in angular spectra were used as a measure for the stability of the chip.

#### 2.2.5. Sensitivity and Limit of Detection Experiment 

NaCl aqueous solutions with different concentrations (0, 1, 2, 3, 4 and 5 g/L) were used for solution with different refractive indexes, and were pumped into the flow cell in turn at a speed of 50 μL/min. 

#### 2.2.6. Immobilization of GAR IgG molecules on SAM-Modified Metal Film

The immobilization process was monitored with angular spectrum setup as shown in Figure 1. After activating the carboxyl groups of MHDA molecules on the silver or gold film with EDC PB buffer solution (10 mg/mL) [39,40,41] for about 60 min at a speed of 30 μL/min, the chip was then washed with PB buffer for ca. 25 min at the same pumping speed. After that, 50 μg/mL GAR IgG solution was pumped in with a speed of 5 μL/min for 1 h and the sensor chip was washed again with PB buffer. Finally, 3% BSA solution was used to block the residue activated carboxyl groups and other active sites to prevent non-specific adsorption. The sensor chips was then ready for the detection of rabbit IgG molecule after washing with PB buffer. 

#### 2.2.7. Detection of Rabbit IgG Molecule 

Different concentrations of rabbit IgG solutions (1, 2, 5, 10, 20, 50 μg/mL) were prepared with 0.01 M PB buffer, and were pumped into the flow cell at a speed of 30 μL/min. Here, a multichannel single cycle kinetics (MC-SCK) method was used to reduce the experiment time, and this method will be more important when the interaction time is longer than 60 min. In our experiment, it takes about 40 min to reach adsorption equilibrium. Thus, two channels were used and channel 1 was flowed with samples with concentrations of 1, 5 and 20 μg/mL, and channel 2 was flowed with samples with concentrations of 2, 10 and 50 μg/mL. It should be noted that this method is suitable only when the nonspecific adsorption could be ignored or a reference channel is introduced.

## 3. Results and Discussion

### 3.1. Optimization and Comparison of Silver and Gold Based SPR Sensors

**Figure 2:**
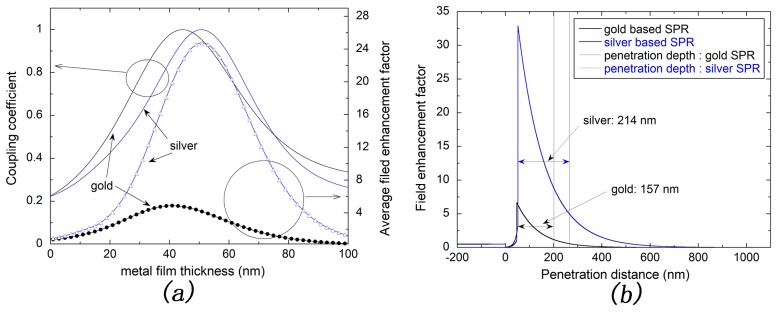
(**a**) Coupling coefficient and average field enhancement factor of gold or silver-based surface plasmon resonance (SPR) sensor with the change of metal film thickness; (**b**) Field enhancement factor versus the penetration distance for silver (50 nm), gold (45 nm).

Silver and gold are normal materials for SPR sensors due to their excellent optical characteristics. In our study, the angular spectrum was calculated using Fresnel theory [42] and the refractive indexes of silver and gold are adopted as 0.0801 + 4.2210i and 0.2201 + 3.2209i [43,44], respectively. Although the adsorption of MUD SAM layer would caught a red shift of the resonance angle of SPR spectrum, a simple three-layer model (glass layer, metal layer and medium layer) without modeling the MUD SAM layer was used. The effect of SAM layer on the comparison of silver- and gold-based SPR sensors could be ignored since it has a low refractive index and an ultra-thin thickness, which are ca. 1.4 and ca. 2 nm, respectively. The coupling coefficient was calculated from the reflectivity in the resonance angle. Additionally, the average field enhancement factor was calculated within 50 nm depth into the water environment, which is enough for detection of large molecules. As shown in Figure 2a, the coupling coefficient of silver-based SPR sensor reaches the maximum at a metal thickness of ca. 50 nm. In addition, the average field enhancement factor also reaches its maximum at nearly the same metal thickness. The sensitivity will be improved when there is more light energy penetrating into the sample solution. Thus, the optimization of silver-based SPR sensor for silver thickness is ca. 50 nm. Similarly, the gold thickness is optimized to ca. 45 nm. 

It is obvious that the average filed enhancement factor of silver-based SPR sensor is nearly five times larger than that of gold-based SPR sensor at the optimized metal thickness as shown in Figure 2a. Further, the filed enhancement factor against the penetration distance was calculated using the matrix theory of electromagnetic field based on Fresnel theory [42]. As shown in Figure 2b, the electric field intensity attenuates exponentially with penetration distance into the solution for either gold or silver-based SPR sensor. In other words, the maximum intensity appears on the metal surface. In addition, the penetration depth into water could be calculated to 214 nm for silver-based SPR sensor and 157 nm for gold-based SPR sensor, at the wavelength of 632.8 nm, according to the formula seen as

(1) δ=λ2π|εm′+εcεc2|12

where λ represents the wavelength of incident light, $\varepsilon_{m}^{\prime }$ is the real part of the dielectric constant of metal material and $\varepsilon_{c}$ expresses the dielectric constant of cover layer. Obviously, in such conditions, the penetration depth of silver-based SPR sensor is deeper than that of gold-based SPR sensor, which is consistent with our calculation, as shown in Figure 2b. Thus, silver-based SPR sensor will present a better optical performance than that of the gold-based sensor.

### 3.2. Stability Test of Silver-Based SPR Sensor

Because of the poor stability of silver, gold-based SPR sensors are more acclaimed in practice. Therefore, the question as to how to make the silver-based SPR sensor more stable in practical use is significantly important. Herein, we used a MUD SAM layer to protect the silver film from being oxidized. 

**Figure 3:**
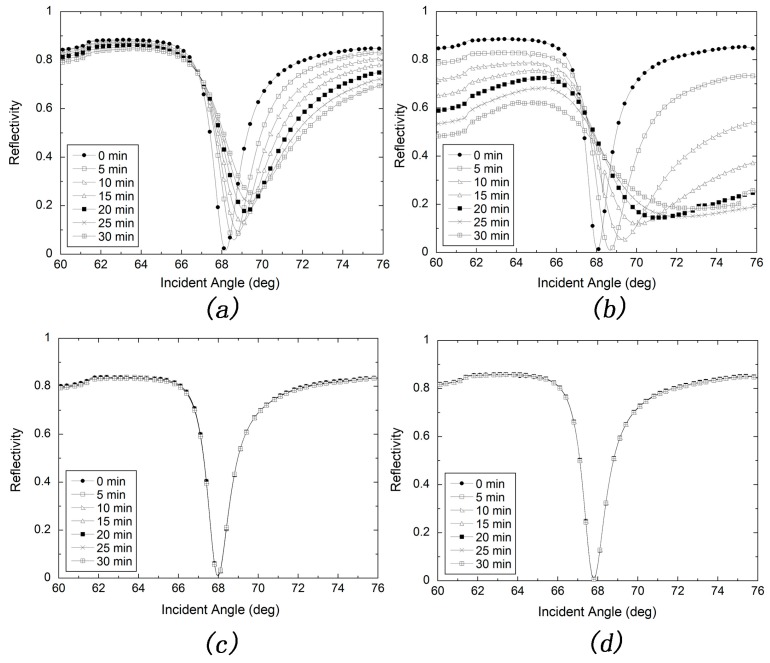
Continuous stability tests of silver-based SPR with a speed of 100 uL/min. (**a**) in water environment without an 11-mercapto-1-undecanol (MUD) self-assembled monolayer (SAM) protection layer; (**b**) in 0.01 M PBS buffer environment without a MUD SAM protection layer; (**c**) in a water environment with a MUD SAM protection layer; (**d**) in 0.01 M PBS buffer environment with a MUD SAM protection layer.

Figure 3a shows the spectrum changes of bare silver-based SPR sensor with a cover layer of water in continuous pumping at a speed of 100 µL/min for 0, 5, 10, 15, 20, 25 and 30 min. The spectrum is obviously broadened and the resonance angle appears to be red shifted, indicating the bare silver film could be easily deteriorated in such condition. In contrast, the silver film protected with SAM could bear such condition as shown in Figure 3c since the spectra obtained at different time remain the same. 

In real biodetection experiments, the bioreagent is normally dissolved in buffer solution, a kind of salt solution, such as 0.01 M PBS buffer. Thus, if the protected silver film could bear salt solution, it will be suitable for practical use in biodetection. Figure 3b shows the spectra of bare silver-based SPR sensor measured with 0.01 M PBS buffer. One can obviously find that the spectrum is broadened more seriously and the red shift of resonance angle is larger than that measured in water. Thus, the bare silver film is more unstable in salt solution. However, the protected silver film remains stable in such conditions, as shown in Figure 3d. All of those results could be ascribed to the protection of SAM layer which has certain thickness and hence some screening effect.

**Figure 4:**
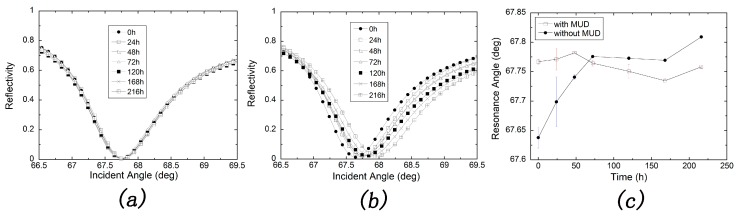
Long-term stability tests of silver-based SPR in ambient environment with (**a**) or without (**b**) protection of MUD SAM layer; (**c**) Resonance angle change with storage time calculated from (**a**,**b**). Error bars represent the standard deviations of three experiments.

We also carried out the comparison of protected silver film and bare silver film for long-term storage in an ambient environment. Keep in mind that the silver film was stayed in water environment only when measuring the angular spectrum. Figure 4a indicates that angular spectra are nearly the same after a storage time of 10 days for SAM-protected silver film. On the contrary, as shown in Figure 4b, the angular spectra represent a red shift like those showed in Figure 3a,b for a bare silver chip. However, one can conclude that the silver film is more stable in ambient environment as the shift is less than that in water or PBS buffer. The resonance angle against storage time was plotted as shown in Figure 4c and those conclusions were more obviously shown in this figure. The little change of resonance angle for the MUD SAM-protected silver-based SPR sensor might result in the artificial error and mechanical error in different tests.

**Figure 5:**
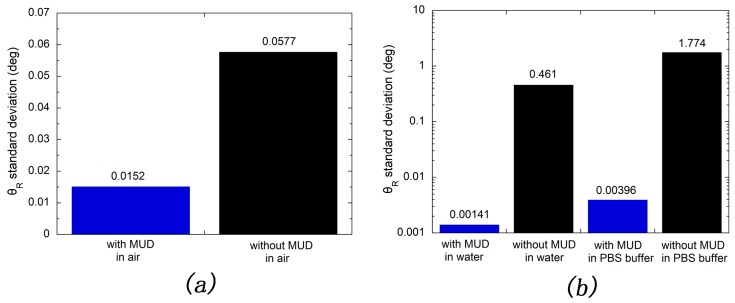
Standard deviations of resonance angle for tests of stability. (**a**) Long-term stability test; (**b**) Continuous stability test.

The standard deviations of resonance angle measured in long-term stability test were calculated and shown in Figure 5a. From which one can obviously find that the resonance angle standard deviation of long-term stored silver-based SPR chip with protection of MUD SAM layer is nearly four times lower than that of which without protection of MUD SAM layer. Further, in continuous experiments, the corresponding multiples of resonance angle standard deviation are 327 and 448 for those measured in water and 0.01 M PBS buffer, respectively, as shown in Figure 5b. One can also obviously find that these multiples are significantly bigger than that obtained in ambient environment. This reason could be that, in a continuous test, the sensor chip would not be disassembled once being installed. Thus, the artificial error was reduced to a very low level. However, the mechanical error existed for all of stability tests. In conclusion, all of those indicate that the stability of silver film is greatly improved with the protection of MUD SAM layer. As shown in Figure 5b, one can also find that silver film are more stable in water than that in 0.01 M PBS buffer. This might be ascribed to the presence of chloride ion, which will attack the silver atom [45]. This suggests that the use of PB buffer rather than PBS buffer is preferable in the long-term detection of rabbit IgG molecule. 

### 3.3. Sensitity Factor (SF) and Limit of Detection (LOD) Tests

The figure of merit (FOM) is an important evaluation factor of SPR sensor [35]. In this paper, the FOM we used is combined sensitivity factor (CSF), which is the product of sensitivity factor (SF) and sensor merit (SM), seen below [46]:

(2) $$\mathrm{CSF}=\mathrm{SF}\times \mathrm{SM}=\frac{\Delta \theta_{R}}{\Delta n}\times \frac{R_{\max}-R_{\min}}{\Gamma },$$

where $\Delta \theta_{R}$ and Δn represent differences of resonance angles and refractive index of covered layers, respectively; Γ expresses the full width at half maximum (FWHM) of angular spectrum.

**Figure 6:**
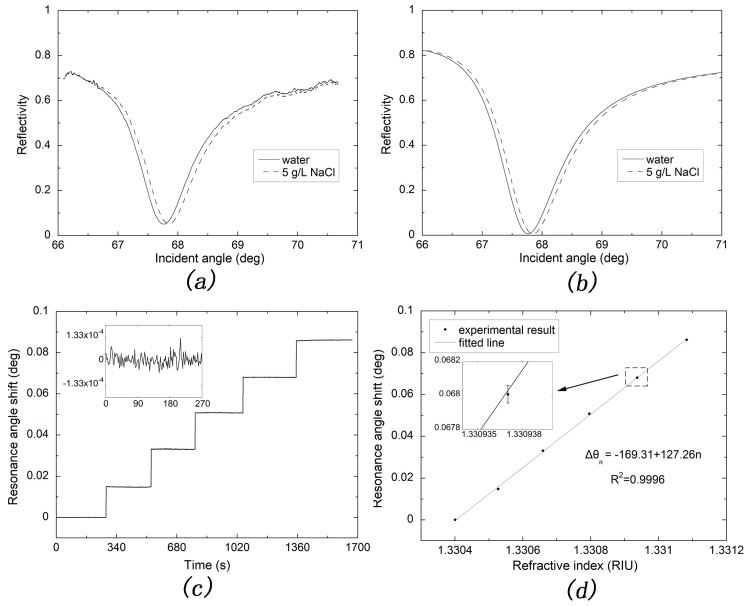
(**a**) Angular spectrum of silver-based SPR sensor measured with water and 5 g/L NaCl solution; (**b**) Angular spectrum of silver-based SPR sensor calculated with water and 5 g/L NaCl solution; (**c**) Resonance angle shift plotted against the experiment time for NaCl solutions (0, 1, 2, 3, 4, 5 g/L ); (**d**) Linear fitting of experiment result from (**c**). Error bars indicate the standard deviations.

Table 1 shows the result calculated by angular spectra obtained with covered layers of water and 5 g/L NaCl solution, as shown in Figure 6a,b. The experimental and theoretical SF of silver-based SPR are calculated to 130.4539 and 115.9590 deg/RIU, respectively, and the corresponding CSF are 84.8342 and 85.7865 RIU^-1^, respectively. The relative relation is in accordance with the reported literature [46].

Figure 6c shows the kinetic curve of NaCl solutions with concentrations of 0, 1, 2, 3, 4, 5 g/L. Moreover, the inset is the water baseline within 270 s. The pump speed was set to 50 µL/min. The refractive index change of NaCl solution compared to water is $\Delta n=1.24947\times 10^{-4}C+2.32129\times 10^{-6}C^{2}$ at the wavelength of 632.8 nm at ambient temperature [47]. The refractive index for water solution was calculated to be 1.3304 according to the total internal reflection angle obtained by pumping water into the flow cell. The resonance angle versus refractive index was then well fitted in linear relationship as seen in Figure 6d. The sensitivity of silver-based SPR sensor is fitted to 127.26 deg/RIU; in addition, one can obviously calculate the refractive index resolution to be $2.207\times 10^{-7}\;\mathrm{RIU}$ with the baseline deviation of $2.808\times 10^{-5}$ deg, as shown in inset of Figure 6c. This result reaches the limit of theoretical resolution of SPR sensor in the wavelength of 632.8 nm predicted in the literature [48]. 

### 3.4. Real-Time Monitoring Biomolecule Interaction

To check the silver-based SPR sensor in real detection of biomolecules. GAR IgG and rabbit IgG were used to study the specific binding of antibody and antigen. 

**Figure 7:**
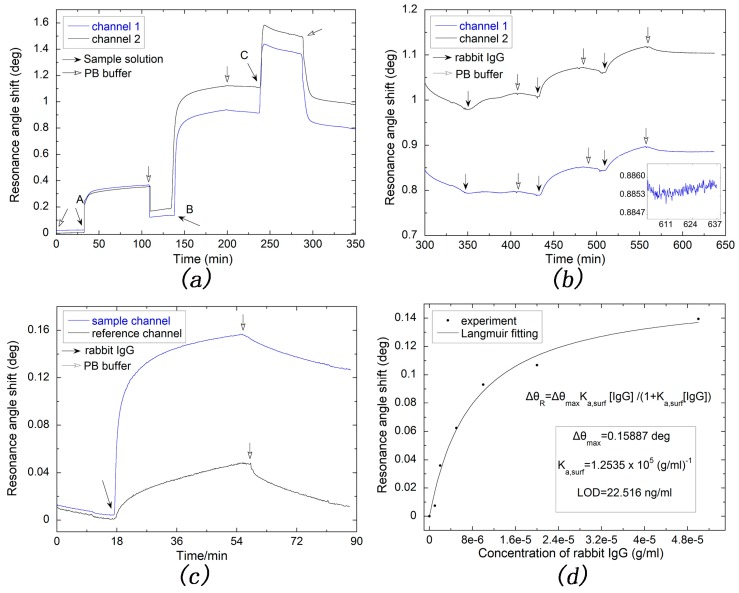
(**a**) Real-time monitoring the modification of silver-based SPR sensor with A is 10 mg/mL EDC solution, B is 50 ug/mL GAR IgG and C is 3% BSA solution; (**b**) The resonance angle shift for detecting different concentrations of rabbit IgG (1, 5, 20 μg/mL for channel 1 and 2, 10, 50 μg/mL for channel 2) and the inset shows resonance angle shift curve after detecting rabbit IgG; (**c**) Test on influence of nonspecific adsorption; (**d**) Langmuir fitting with data measured from (**b**).

Figure 7a shows the detailed process of modifying silver-based SPR sensor chip. The curves of channel 1 and channel 2 are nearly identical since the modification process are the same. Thus, hereinafter, we simply need to analyze the curve of channel 1. After pumping with 10 mg/mL EDC solution for about 1 h, the shift of resonance angle is ca. 0.1 deg, which indicates that the carboxyl group is successfully activated. And the GAR IgG molecule also successfully attached onto the silver sensor chip since a larger resonance angle shift was observed and the binding affinity is strong since the resonance angle comes back little after washing with 0.01 M PB buffer for at least 30 min. However, the resonance angle shifted back slightly when 3% BSA solution was continuously pumping in. The reason for this phenomenon could be ascribed to that BSA molecules replaced some GAR IgG molecules which are nonspecific adsorbed.

As shown in Figure 7b, two channels were pumped with different concentrations of rabbit IgG solutions. This MC-SCK method not only reduces the experiment time but also makes the rising trend more evident due to the larger difference of concentrations in every channel than that using just one channel for detecting the same number of samples. 

For studying the influence of nonspecific adsorption, a reference channel was used. After activating the carboxyl group, the sample channel was pumped into 50 μg/mL GAR IgG solution, and the reference channel was still pumped into PB buffer. Then, both channels were blocked with 3% BSA solution before 30 μg/mL rabbit IgG solution was pumped in. As shown in Figure 7c, when the adsorption reaches the dynamic equilibrium, the angle shift of sample channel is 0.153 deg and reduces to 0.123 deg after washing with PB buffer for 30 min, which are 3.2 times and 11.6 times larger than those of the reference channel, in which the corresponding data are 0.0475 and 0.0106 deg. The former decreased by 19.61% while that of the latter is 77.68%, indicating the specific adsorption in the sample channel is overwhelming, while the binding affinity in the reference channel is much weaker due to its nonspecific adsorption nature. It is therefore reasonable to ignore the nonspecific adsorption in the sample channel and adopt the MC-SCK method as described above.

Figure 7d shows a Langmuir fitting of MC-SCK data obtained at the dynamic adsorption equilibrium, according to the 1:1 binding model of Langmuir adsorption, expressed as [49,50]:

(3) $$\frac{\Delta \theta_{R}}{\Delta \theta_{\max}}=\frac{K_{a,\mathrm{surf}}\left[\mathrm{RIgG}\right]}{1+K_{a,\mathrm{surf}}\left[\mathrm{RIgG}\right]}\;$$

where $\Delta \theta_{R}$ is the resonance angle shift, $\Delta \theta_{\max}$ is the maximum resonance angle shift, $K_{a,\;\mathrm{surf}}$ is the surface-confined thermodynamic affinity constant and [RIgG] is the concentration of rabbit IgG solution.

According to our Langmuir fitting, $\Delta \theta_{\max}$ is 0.15887 deg and the $K_{a,\;\mathrm{surf}}$ is $1.2535\times 10^{5}\;\mathrm{mL}/g$, that is $1.8803\times 10^{7}\;M^{-1}$, which is consistent with the binding affinity reported in those literature [51,52,53]. A standard deviation of $1.4904\times 10^{-4}$ deg was obtained with 0.01 M PB buffer, as shown in inset of Figure 7b. Taking the detection limit as three times this value, the LOD of our silver-based SPR sensor for rabbit IgG is thus calculated to be 22.516 ng/mL. This value is much improved compared to those obtained with an optical fiber sensor [54] and a surface plasmon resonance imaging platform [55], which are 70 ng/mL and 1.33 nM, respectively. 

**Figure 8:**
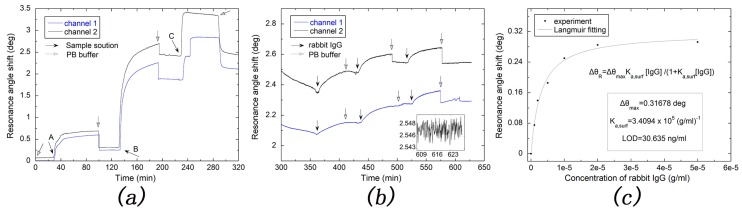
(**a**) Real-time monitoring the modification of the gold-based SPR sensor with A is 10 mg/mL EDC solution, B is 50 ug/mL GAR IgG and C is 3% BSA solution; (**b**) The resonance angle shift for detecting different concentrations of rabbit IgG (1, 5, 20 ug/mL for channel 1 and 2, 10, 50 ug/mL for channel 2) and the inset shows resonance angle shift curve after detecting rabbit IgG; (**c**) Langmuir fitting with data measured from (**b**).

For comparison with the gold-based SPR sensor, the detection of rabbit IgG for the gold-based SPR sensor was also carried out with the same condition and procedure. As shown in Figure 8a,b, kinetic adsorption curves obtained by the gold-based SPR chip were similar with those obtained by silver-based SPR chip. As shown in Figure 8c, for the gold-based SPR sensor, $\Delta \theta_{\max}$ and $K_{a,\;\mathrm{surf}}$ are 0.31678 deg and $3.4094\times 10^{5}\;\mathrm{mL}/g$, respectively, which are all larger than those of the silver-based SPR sensor. The LOD of the gold-based SPR sensor chip for rabbit IgG is also calculated to be 30.635 ng/mL with a standard deviation of $1.101\times 10^{-3}$ deg. Thus, our silver-based SPR sensor showed lower LOD and higher stability for rabbit IgG detection than the gold-based SPR sensor. 

## 4. Conclusions

In this paper, we make a comparison of SPR sensor based on silver film and gold film. The former has a better performance in the field enhancement and penetration depth, which is 5 times and 1.4 times higher than that of the gold film-based sensor, respectively. To overcome the instability of silver film and investigate the efficiency of the protected layer, a SAM of MUD was used as the protecting layer, and the results show that the protected silver film exhibited a perfect SPR angular spectrum either in water or in 0.01 M PBS solution, or after long-term storage in an ambient environment. On the contrary, the bare silver film are seriously deteriorated even in water for less than 10 min. Using the SAM-protected silver film for the SPR sensor, the sensitivity factor (SF) and combined sensitivity factor (CSF) were measured to be 130.4539 deg/RIU and 84.8342 ${\mathrm{RIU}}^{-1}$, respectively. While the corresponding theoretical values are 115.9590 deg/RIU and 85.7865 ${\mathrm{RIU}}^{-1}$. In addition, the refractive index sensitivity was calculated to 127.26 deg/RIU, and the refractive index resolution as high as $2.207\times 10^{-7}\;\mathrm{RIU}$ was obtained. As an immunosensor, an LOD as low as 22.516 ng/mL for rabbit IgG was achieved.

## Figures and Tables

**Table 1:** The characteristics of silver-based SPR sensor measured with water and 5 g/L NaCl solution. Sensitivity factor (CSF); sensitivity factor (SF); sensor merit (SM).

Silver-based SPR	*SF* ($deg\cdot RIU^{-1}$)	SM ($deg^{-1}$)	CSF ($RIU^{-1}$)
Experimental data	130.4539	0.6503	84.8342
Theoretical value	115.959	0.7398	85.7865
